# Structural and functional characterization of cold-active sialidase isolated from Antarctic fungus *Penicillium griseofulvum* P29

**DOI:** 10.1016/j.bbrep.2023.101610

**Published:** 2023-12-20

**Authors:** Aleksandar Dolashki, Radoslav Abrashev, Dimitar Kaynarov, Ekaterina Krumova, Lyudmila Velkova, Rumyana Eneva, Stefan Engibarov, Yana Gocheva, Jeny Miteva-Staleva, Vladislava Dishliyska, Boryana Spasova, Maria Angelova, Pavlina Dolashka

**Affiliations:** a Institute of Organic Chemistry with Centre of Phytochemistry, Bulgarian Academy of Sciences, Sofia, 1113, Acad. Georgy Bonchev str., bl. 9, Bulgaria; b The Stephan Angeloff Institute of Microbiology, Bulgarian Academy of Sciences, Sofia, 1113, Acad. G. Bonchev str., bl. 26, Bulgaria

**Keywords:** Antarctic fungi, Sialidase, Cold-active enzyme, Structural characterization, 3D-structure, Fluorescence analysis, pH-stability

## Abstract

The fungal strain, *Penicillium griseofulvum* P29, isolated from a soil sample taken from Terra Nova Bay, Antarctica, was found to be a good producer of sialidase (P29). The present study was focused on the purification and structural characterization of the enzyme. P29 enzyme was purified using a Q-Sepharose column and fast performance liquid chromatography separation on a Mono Q column. The determined molecular mass of the purified enzyme of 40 kDa by SDS-PAGE and 39924.40 Da by matrix desorption/ionization mass spectrometry (MALDI-TOF/MS) analysis correlated well with the calculated mass (39903.75 kDa) from the amino acid sequence of the enzyme. P29 sialidase shows a temperature optimum of 37 °C and low-temperature stability, confirming its cold-active nature. The enzyme is more active towards α(2 → 3) sialyl linkages than those containing α(2 → 6) linkages.

Based on the determined amino acid sequence and 3D structural modeling, a 3D model of P29 sialidase was presented, and the properties of the enzyme were explained. The conformational stability of the enzyme was followed by fluorescence spectroscopy, and the new enzyme was found to be conformationally stable in the neutral pH range of pH 6 to pH 9. In addition, the enzyme was more stable in an alkaline environment than in an acidic environment. The purified cold-active enzyme is the only sialidase produced and characterized from Antarctic fungi to date.

## Highlights

• The purified cold-active enzyme sialidase is isolated from fungal strain, *Penicillium griseofulvum* P29, isolated from a soil sample from Terra Nova Bay, Antarctic.
• The molecular mass of the purified enzyme is 39924.40 Da.
• The enzyme is more active towards α(2. → 3) sialyl linkages than those containing α(2 → 6) linkages.
• The new enzyme is conformationally stable in the neutral pH range of pH 6 to pH 9.

## 1 Introduction

Antarctica is a land of extremes – it is the highest, whitest, driest, and windiest continent on Earth. Should be added here water availability and precipitation, continuous freeze-thaw cycles, and high UV incidence. It is also unbelievably cold and experiences long periods of darkness. The average annual temperature ranges from about −10 °C on the coast to −60 °C at the highest parts of the interior [1,2]. The lowest temperature ever recorded on the Earth's surface was −89.2 °C at Vostok station on July 21, 1983. Moreover, the snow surface temperature on the Eastern Antarctic Plateau regularly reaches −98 °C during the polar winter night. This complex set of environmental conditions makes life difficult for animals as well as plants but many microorganisms can thrive here [2,3]. Microbial life is mainly represented by archaea, bacteria, and fungi that play great ecological roles in the Antarctic environment [[4], [5], [6]]. Although fungi occur in various types of living and non-living substrates in different Antarctic niches, knowledge of the diversity, ecological significance, and biotechnological applications of Antarctic fungi remains scarce [7,8].

Cold-adapted microorganisms possess a variety of mechanisms to maintain vital cellular functions at low temperatures, such as cold-active extracellular enzyme production, synthesis of antifreeze protein, elevated unsaturated fatty acids, an increase of pigmentation, resistance to osmotic stress, etc. [9,10]. Cold-active enzymes can catalyze different reactions at low temperatures because of their high specific activity and capability to support transcription and translation at low temperatures [11,12]. These naturally evolved enzymes are very useful in many biotechnological applications requiring high activity at mild temperatures or fast heat-inactivation rate [13]. Cold-active enzymes are nowadays in great demand in food, detergent, paper, textiles, medicine, pharmacy, cosmetics, and organic chemical synthesis, due to their high efficiency and environmental friendliness [14]. Examples of cold-active enzymes currently being used in biotechnological and industrial applications are cellulase, amylase, inulinase, protease, isomerase, xylanase, chitinase, lipase, superoxide dismutase, catalase [7,9,[15], [16], [17], [18], [19]].

Despite of intensive search for cold-active enzymes, there are no reports of Antarctic microbial (bacterial or fungal) producers of sialidase. Sialidases (neuraminidases, N-acetylneuraminic acid hydrolases, EC 3.2.1.18) are the enzyme family of glycohydrolytic enzymes that remove sialic acid residues from various sialoderivatives [20]. They hydrolyze terminal N- or O-acylneuraminic acids, which are α(2,3)-, α(2,6)-, α(2,8)- or α(2,9)-linked to glycoproteins, glycolipids, polysaccharides, mucopolysaccharides, and oligosaccharides. Most sialidases are exosialidases that hydrolyze the sialic substrates with terminal sialic acids, but fewer are endosialidases. These enzymes are widely distributed in nature and have been identified in numerous viruses, protozoa, bacteria, vertebrates, and other animals [21].

Sialidases play a pivotal role in the infection process and pathogenesis covering nutrition for bacteria and promoting bacterial colonization, bacterial adhesion, bacterial internalization, biofilm formation, and the binding of toxins to host cells [22,23]. Purified sialidase preparations have been applied to analyze the glycan structure in many biomedical experiments, for the production of bioactive compounds as sialoglycoproteins and sialyloligosaccharides, to participation in regioselective hydrolysis reactions, and for the design of new drugs and therapies [[24], [25], [26]]. There is a continuing interest in finding a new sialidase, especially a sialidase from nonvirulent fungi for applications in food and pharma industries since secreted enzymes can be easily overexpressed and purified in large quantities from a fungal culture [27,28].

The reports for sialidase synthesis by fungi are very rare. Published data refer only to pathogenic fungi including *Candida albicans* isolated from a human vaginal specimen [29] and *Aspergillus fumigatus* [30,31]. Van Djik et al. (2011) have patented sialidase production by *Penicillium chrysogenum* Dierckx, ATCC® 32794™. Recently, we presented a detailed study on the distribution of sialidases among 113 fungal strains from non-clinical isolates [32]. Enzyme activity was found in culture filtrates from 77 strains, although the sialidase gene was identified in sialidase-positive and sialidase-negative strains. Thirty-four tested strains have been isolated from Antarctica. Among them, 30 demonstrated sialidase activity as a clear habitat-dependent response. The strain *P. griseofulvum* Р29 proved as the most promising producer of this enzyme after optimization of culture conditions.

The present study reports the purification of a novel sialidase from the fungus *P. griseofulvum* P29, its structural characteristic, and biochemical properties. So far in the literature are not found reports on purification and characterization of sialidase from Antarctic origin.

## 2 Materials and methods

### 2.1 Fungal strain and culture conditions

The fungal strain *P. griseofulvum* Р29 (with optimal growth temperature of 25 °C), isolated from a soil sample taken from Terra Nova Bay, Antarctica [32] was used for the experiments. The strain has been deposited in National Bank for Industrial Microorganisms and Cell Cultures, Bulgaria (NBIMCC 9106). Long-term preservation was carried out in the Microbank system (Prolab Diagnostics, Richmond Hill, Canada) consisting of sterile vials that contain 25 porous, colored beads and a cryopreservative fluid at −80 °C. Before use, the conidiospores were grown on Beer agar at 28 °C for 7 days.

Cultivation was performed in a 3 L bioreactor ABR-09 developed and constructed by the former Central Laboratory for Bioinstrumentation and Automatisation (CLBA) of the Bulgarian Academy of Sciences. The bioreactor was equipped with temperature, pH, and dissolved oxygen (DO) automatic monitoring equipment and a control system. For sialidase production Wh medium, as described previously [32] was used. Cultivation was performed in two stages. For the first stage, 74 mL of Wh medium were inoculated with 6 ml spore suspension at a concentration of 2x10^8^ spores/mL in 500 mL Erlenmeyer flasks at 25 °C for 24 h on a rotary shaker (220 r.p.m.). For the second stage, 200 mL of the 1-stage culture was brought into the 3 L bioreactor, containing 1800 mL of the Wh medium. The cultures were grown at 20 °C with a stirrer speed of 400 rpm and airflow of 0.5 v.v.m for 48 h.

### 2.2 Enzyme activity determination

Sialidase activity was measured quantitatively by colorimetric determination of free sialic acid by the thiobarbituric acid method of Uchida et al. [33]. One unit of enzyme activity was defined as the amount that releases 1 μmol of N-acetyl-neuraminic acid for 1 min at 37 °C using glycomacropeptide (Bulgarian Patent № 47647/IIR, Abrashev et al. [34]) as a substrate. The protein content of the enzyme preparations was determined by the procedure of Lowry et al. [35].

### 2.3 Purification of sialidase

Mycelia were removed from 48-h culture liquid by filtration to obtain a cell-free solution. To ensure the supernatant was completely cell-free, it was filtered through Celite and Millipore's device Pellicon XL Durapore 0.1, concentrated, and fractionated by ultrafiltration with Pellicon XL 10 (10 kDa). All purification steps were carried out at 4 °C using an FPLC system (ÄKTA purifier GE Healthcare Life Sciences, USA). The filtrate containing crude enzyme sialidase was brought to 40 % ammonium sulfate saturation. The dialyzed enzyme solution was concentrated and loaded onto a Q-Sepharose column pre-equilibrated with 0.01–0.2 M potassium phosphate buffer at pH 7.8. Enzyme protein was eluted with the same buffer 0.02 M with pH 7.8. At each stage of purification, active fractions were analyzed for sialidase activity.

### 2.4 Determination of purity and molecular weight of P29 sialidase

The purity and molecular weight (Mw) of the isolated P29 sialidase were determined using two methods: electrophoretically by 12 % sodium dodecyl sulfate-polyacrylamide gel electrophoresis (SDS-PAGE) and by MALDI-TOF-TOF mass spectrometric analysis on an AutoflexTM III (Bruker Daltonics). The crude sialidase enzyme and the resulting pure enzyme after a Q-Sepharose column were applied to a 12 % SDS-PAGE comprising a 5 % loading gel and a 12 % separating gel, according to the Laemmli method with modifications [36]. N,N,N′,N′-tetramethylethylenediamine (TEMED), DL-dithiothreitol acrylamide/bis-acrylamide (30 % solution), bromophenol blue sodium salt (Sigma-Aldrich, Germany) and ammonium persulfate (APS) (GE Healthcare, Sweden) were used for SDS electrophoresis. Equal volumes of 20 μg of the samples dissolved in Laemmli sample buffer (Tris/HCl pH 6.8, glycerol 20 %, SDS 4 %, and bromophenol blue 0.02 %) were applied to the gel. A protein standard mixture of eight proteins (SERVA Unstained SDS PAGE Protein Marker 6.5–200 kDa Liquid Mix, Germany) was also applied. Proteins in the samples were separated by 12.0 % SDS-PAGE and visualized by staining with Coomassie Brilliant Blue G-250.

The molecular weight of the sialidase enzyme was determined according to the Mw ranging from 6.5 to 200 kDa of the marker. The molecular masses of the isolated fractions were measured by Autoflex™III, a high-performance MALDI-TOF & TOF/TOF system (Bruker Daltonics) that uses a 200 Hz tripled Nd–YAG laser operating at a wavelength of 355 nm. The assay was performed by mixing 2.0 μl of the sample with 2.0 μl of matrix solution (7 mg/ml α-cyano-4-hydroxycinnamic acid (CHCA) in 50 % CN containing 0.1 % TFA). 1.0 μl of the mixture was applied to a 192-well stainless steel target, dried at room temperature (20 °C), then subjected to mass analysis. A total of 3500 shots were received in MS mode and an impact energy of 4200 was applied.

### *2.5* *Identification of isolated enzyme from fungal strain P. griseofulvum* P29

An isolated enzyme from the fungal strain *P. griseofulvum* P29 was identified by 12 % SDS-PAGE [36]. The electrophoresis protein band corresponding to sialidase was excised with a scalpel and washed twice with 150 μL of 200 mmol/L ammonium bicarbonate in 50 % ACN/MQ (30 min at 30 °C). After destaining, the gel was dried in a SpeedVac (Thermo Savant, Holbrook, NY) [37] and subjected to tryptic digestion with porcine trypsin (Madison, WI, USA) as previously described [37].

The resulting peptides, after enzyme digestion with trypsin, were analyzed by MALDI-TOF-TOF mass spectrometry on AutoflexTM III, and their molecular masses were determined. Analysis was performed with a high-throughput MALDI-TOF& TOF/TOF system (Bruker Daltonics, Bremen, Germany), at a wavelength of 355 nm, after mixing 1.0 μL of the sample with 1.0 μL of matrix solution (8 mg/mL α-cyano-4-hydroxycinnamic acid (CHCA) in 50 % ACN containing 0.1 % TFA). Dropped samples on a 192-well plate, after drying at room temperature, were imaged in MS mode with a collision energy of 4200 applied. The mass spectrometer was calibrated with a standard mixture of peptides. The amino acid sequences (AAS) of the peptides were identified from MS/MS spectra performed in reflector mode with external calibration with Glu-fibrin-peptide B fragments.

### 2.6 3D-structure of sialidase from *P. griseofulvum* P29

Comparative amino acid sequence analysis of the purified sialidase from *P. griseofulvum* P29 with other 6 sialidases was conducted using the Clustal Omega multiple sequence alignment program, after alignments between their sequences [38]. Evolutionary relationships can be seen via viewing Cladograms or Phylograms. The highest homology of the sialidase enzyme from *P. griseofulvum* P29 was found with the isolated sialidase enzyme from *Penicillium patulum* (*P. griseofulvum*) (A0A7S6VMA0_PENPA). Therefore, the 3D model of *P. griseofulvum* P29 sialidase was built based on the 3D structure of *P. patulum* sialidase after applying several programs, such as Swiss-Prot, BLAST, and RasWin. Additional graphical processing with the Swiss-Pdb Viewer for homology modeling of the enzyme structure was applied.

### 2.7 Biochemical characterization of the sialidase from *P. griseofulvum* P29

#### 2.7.1 Effect of temperature and pH on P29 sialidase activity

The optimum temperature of P29 sialidase was examined at temperatures of 5, 20, 25, 30, 35, 37, 40, 45, 50, 60, and 70 °C. Thermal stability of the enzyme was determined by pre-incubating enzyme samples at 20, 37, and 40 °C for different time intervals (1, 6, 18, and 24 h). Glycomacropeptide was used as a substrate in a concentration of 10 mg/ml in 0,1 M phosphate buffer pH 5,8. Each sample was assayed at the standard condition for sialidase analysis - 37 °C. A control sample was assayed without pre-incubation and assumed to have 100 % activity.

The optimum pH of P29 sialidase was studied over a pH range from 3.0 to 9.0 using the following buffer types: 0.1 M citrate buffer (3.0–6.0), 0.01 M phosphate buffer (6.0–8.0) and 0.05 M tris-maleate buffer (7.7–9.0). Glycomacropeptide was used as a substrate in a concentration of 10 mg/ml in each buffer used for the respective pH value. An amount of 100 μL of glycomacropeptide solution in each buffer of a certain pH was mixed with the same volume of sialidase preparation. The mixtures were assayed according to the procedure of Uchida et al. [33]. The relative activity was calculated considering the maximum activity as 100 %.

#### 2.7.2 Sialidase substrate specificity assays

The substrate specificity of the extracellular sialidase of *P. griseofulvum* P29 was examined with the following compounds, suspended in 0.1 M citrate buffer, pH 4.0: α(2 → 3) and α(2 → 6) sialylactose sodium salt (BLD Pharmatech, Shanghai, China), human and bovine transferrin, horse serum (Sigma-Aldrich Chemie GmbH, Steinheim, Germany), colominic acid (Koch-Light, Colnbrook Berks, England), fetuin (Serva, Heidelberg, Germany), gangliosides GM1, GM2 (Sigma Chemical CO, USA), gangliosides from bovine brain (Fluka BioChemika, Switzerland), and GMP. The gangliosides were suspended in absolute methanol with or without addition of 1 % Triton X-100. The concentration of substrates was adjusted to 0,15 mM sialic acid content by estimation of the free sialic acids released from each substrate after acid hydrolysis of the glycoproteins. Sialic acid content of glycolipids was adjusted according to their commercial descriptions. All sialidase assays were carried out at 37 °C in triplicate.

#### 2.7.3 Fluorescence analysis of the structure and pH-stability of sialidase P29

Fluorescence spectra of the purified enzyme (0.05 mg/ml) with absorbance < 0.05 were recorded at an excitation wavelength (*λ*_ex_) of 295 nm on a Shimadzu RF-6000 spectrofluorometer using a xenon lamp as the excitation source. The conformational pH stability of sialidase was investigated over a wide pH range of the acidic and alkaline region by capillary microtitration with 0.5 N HCl or 0.5 N NaOH. After incubating the enzyme for 10 min at each pH value, emission spectra were recorded in the range of 310 nm–500 nm, after excitation of the samples at *λ*_ex_ 295 nm, at 25 °C. The result of three measurements at the same conditions is reported.

#### 2.7.4 Statistical evaluation of the results

All results presented in this study were obtained and evaluated from experiments with at least three replicate using three parallel runs. Statistical evaluation of results was performed by Student's *t*-test (*t*-test) for MIE (mean interval estimate), analysis of variance (ANOVA), and Dunnet's post test, with a significance level of 0.05.

## 3 Results

### 3.1 Purification of P. griseofulvum sialidase P29

The sialidase enzyme was produced by the fungal strain *P. griseofulvum* P29, at a temperature of 25 °C for 48 h. Cell-free culture filtrate (600 ml) was used in experiments to obtain pure sialidase enzyme. The enzyme purification protocol involves three steps: concentration of the filtrate by ultrafiltration, precipitation of the enzyme with (NH_4_)_2_SO_4_, followed by purification of the homogeneous enzyme by anion exchange chromatography on Q-Sepharose. The summarized steps of the purification procedures are presented in Table 1.

**Table 1:** Summary of the purification process of sialidase from *P. griseofulvum* P29.

Purification step	Total activity (U)	Total protein (mg)	Specific activity (U/mg protein)	Purification (fold)	Yield (%)
Culture filtrate	3600	397.2	9.10	1.0	100.0
Ultrafiltration with Pellicon XL10	985	85.04	24.52	2.7	27.4
Precipitation with **(NH**_**4**_**)**_**2**_**SO**_**4**_	497	51.62	41.69	4.6	13.8
Q Sepharose	332	1.8	184.20	20.2	9.2

In the first step of purification, the collected supernatant with 9.10 U/mg specific activity was subjected to ultrafiltration with Pellicon XL 10 (10 kDa). The procedure makes it possible to achieve a 2.7-fold increase in specific activity and an enzyme yield of 27.4 %. Further purification of P29 sialidase was achieved by (NH_4_)_2_SO_4_ precipitation, resulting in a 4.7-fold increase in specific activity at 13.8 % enzyme yield.

**Fig. 1:**
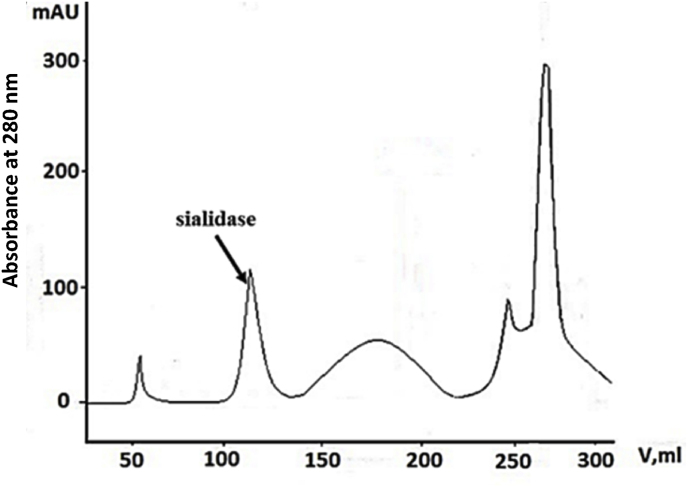
Chromatographic patterns observed during purification of *P. griseofulvum* sialidase applied to a column of Q-Sepharose, using an FPLC system. The column was pre-equilibrated with 0.01–0.2 M potassium phosphate buffer at pH 7.8 and the enzyme was eluted with the same buffer.

The final stage of the scheme involves liquid chromatography - elution of the enzyme on Q-Sepharose (Fig. 1), after which the specific sialidase activity reaches 184.2 U/mg protein, which represents a purification factor of 20.2 times and a 9.2 % yield.

### 3.2 Characterisation of the isolated enzyme from fungal strain *P. griseofulvum* P29

#### 3.2.1 Identification and determination of the molecular weight of the isolated enzyme

**Fig. 2:**
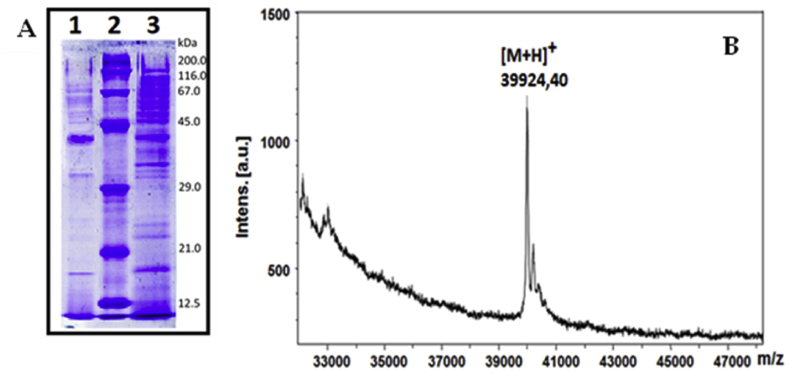
Molecular weight of a sialidase from the fungal strain *P*. *griseofulvum* P29, determined by: A) 12 % SDS-PAGE analysis (Position 1 –purified enzyme on Q-Sepharose; position 2 – standard; position 3 – crude extract); B) MS analyses of purified enzyme on a Q-Sepharose column.

Two methods were applied to identify and determine the Mw of the isolated enzyme sialidase from the fungal strain *P. griseofulvum* P29. The purity of the isolated enzyme P29 was confirmed by 12 % SDS-PAGE analysis (Fig. 2A), wherein in the crude extract (position 3) many bands are observed, which decrease after column purification and reach mainly one band (position 1).

The molecular mass of the enzyme was found to be about 40 kDa relative to the electrophoresis standard (position 2). The result of SDS-PAGE analysis presented in Fig. 2A, indicates the presence of minor contamination. Therefore, the sample corresponding of position 1 was rechromatographed under the same conditions on Q-Sepharose by FPLC. The obtained result after repeated SDS-PAGE showed the presence of one band at 40 kDa corresponding to the purified enzyme. The other method involves precision mass spectrometric analysis performed by MALDI-Tof/MS.

From the MS spectrum shown in Fig. 2B, the exact molecular mass of 39924.40 Da was determined, which correlated well with the calculated mass (39903.75 kDa) from the amino acid sequence of the enzyme (Table 2).

**Table 2:** Amino acid sequences (AASs) of peptides obtained after tryptic digestion of the enzyme and determination by analysis of their MS/MS spectra.

AAS of Peptide	Mass Exp. [M+H]+
1. GPDGPTHFR	983.46
2. SLCVAKSTDNGR	1250.69
3. EALLMVFETTR	1309.72
4. GPDGPTHFRITVCR	1555.81
5. TIDNGREALLMVFETTR	1966.12

#### 3.2.2 Primary structure of P29 sialidase

**Fig. 3:**
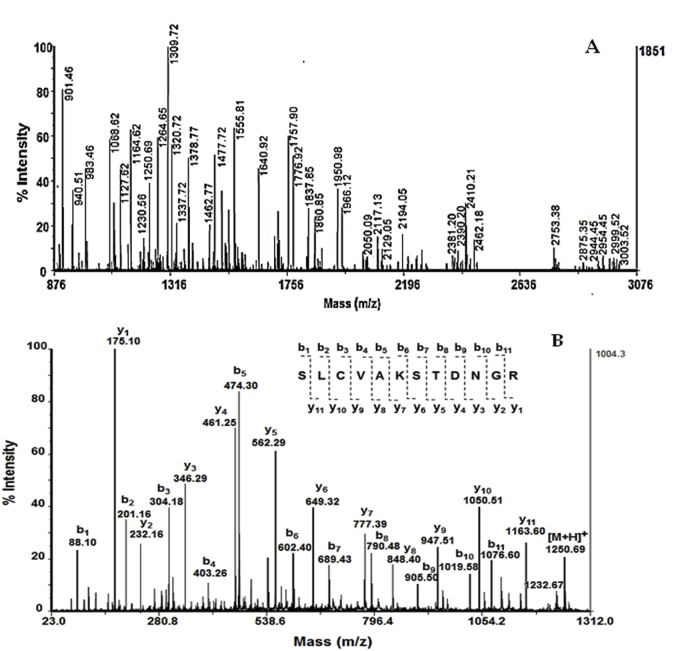
Mass spectrometric analysis of Р29 sialidase: A) MS spectrum of peptides obtained after tryptic digestion of enzyme; B) MS/MS spectrum and identified ААS of peptide [M+H]^+^ at *m*/*z* 1250.69.

Further evidence that the isolated enzyme from fungal strain *P*. *griseofulvum* P29 is a sialidase was determined partial amino acid sequences by mass spectrometric analyses. This was achieved after analysis of the protein band at position 1 on 12 % SDS-PAGE, which corresponds in Mw to the enzyme sialidase. After cutting and destaining the gel, and subjected to enzymatic digestion with porcine trypsin, the molecular masses of the resulting peptides were determined from the MS spectrum measured by MALDI-TOF-TOF mass spectrometry on an AutoflexTM III (Fig. 3A). The AAS of these peptides were identified from MS/MS spectra performed in reflecting mode (Fig. 3B).

Following the MS/MS spectrum of the fragmented y- and b-ions of peptide [M+H]^+^ at *m*/*z* 1250.69 presented in Fig. 3B, the AAS of the peptide was determined. Applying the same approach, the AAS of 6 peptides [M+H]^+^ at *m*/*z* 901.46; *m*/*z* 983.46; *m*/*z* 1250.69; *m*/*z* 1309.72; *m*/*z* 1555.81 and *m*/*z* 1966.12), presented in Table 2 were determined.

The results of MS/MS spectra and determined AAS from mass spectrometric analyzes of these peptides are homologous to the published amino acid sequence determined by sequencing the genome of the fungal strain P29 sialidase. This is further evidence that the enzyme isolated from the *P. griseofulvum* P29 fungal strain is a sialidase.

The high homology of the AAS of the purified P29 enzyme with the AAS of 6 sialidases from other fungal strains was determined using (https://www.ebi.ac.uk/Tools/msa/clustalo/) the Clustal Omega program.

Calculated identity of AAS of sialidase from *P. griseofulvum* is very high with *Neuraminidase P. griseofulvum* [P*29* KXG51741.1] - 96.1 % identity; (98.3 % similar) in 362 aa overlap; *P. rubens Wisconsin* 54-1255 [XP_002560442.1 Pc16g00170] - 90.9 % identity; (96.7 % similar) in 363 aa overlap; *P. camemberti* [CRL20054.1] - 91.8 % identity; (96.4 % similar) in 364 aa overlap; *P. italicum* [KGO67483.1] - 90.7 % identity; (95.9 % similar) in 364 aa overlap; *A. fumigatus Af293* [XP_748867.1] - 66.8 % identity (84.6 % similar) in 364 aa overlap and Sialidase *A. egyptiacus* [KAI9376722.1] *-* 64.8 % identity (83.3 % similar) in 366 aa overlap (Table 3).

**Table 3:**
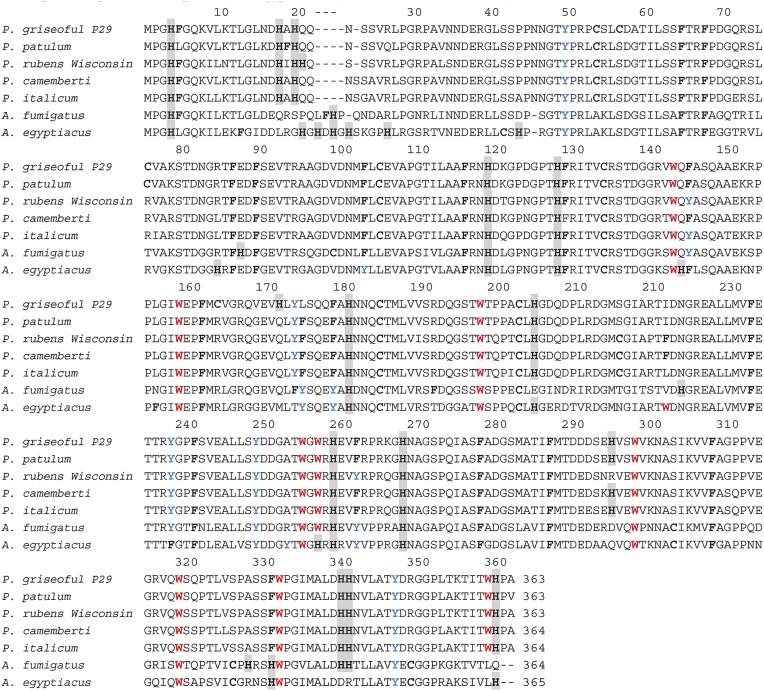
Sequence alignment of sialidase from *P. griseofulvum* P29 with sialidases from *P. patulum* (KXG51741.1 Neuraminidase)*, P. rubens Wisconsin* (XP_002560442.1 Pc16g00170)*, P. camemberti* (CRL20054.1 Neuraminidase) *P. italicum* (KGO67483.1 Neuraminidase), *A. fumigatus* (XP_748867.1 BNR/Asp-box repeat domain protein) *A. egyptiacus* (K AI9376722.1 Sialidase).

### 3.3 Biochemical characterization of the enzyme

#### 3.3.1 Determination of temperature dependence of enzyme activity

**Fig. 4:**
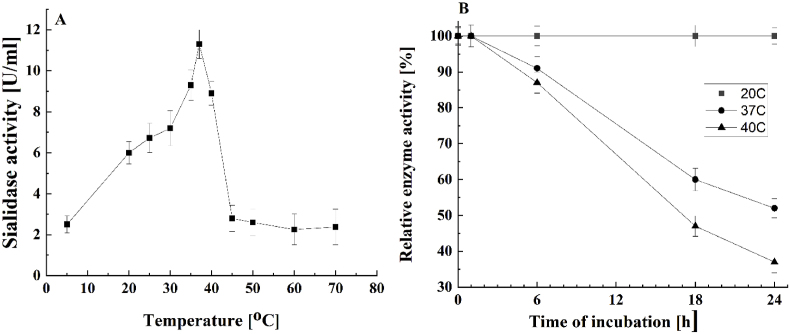
Temperature dependency of the *P. griseofulvum* sialidase activity (see M&M). (A) temperature-dependent profiles; (B) thermostability. Values are means of three replicates; bars represent the standard deviation. Temperature turns out statistically significant effect on the enzyme activity (p ≤ 0.05).

The activity of the purified sialidase from Antarctic *P. griseofulvum* was determined at different temperatures (Fig. 4). The enzyme activity showed a continuous increase with the increase in temperature from 5 °C to 37 °C with an optimum at 37 °C. It retained nearly 20 % of the activity at 5 °C and about 50 % of the activity at 20 °C.

Further, the increase in incubation temperature above 37 °C sharply decreased the enzyme activity, with approximately 78.8 % and 24.8 % of activity being retained at 40 °C and 45 °C, respectively, concerning 100 % at 37 °C (Fig. 4A).

Isolated enzyme *P. griseofulvum* sialidase demonstrated high stability at low temperatures: the incubation at 20 °C, 37 °C, and 40 °C for 1 h retained 100 % of activity (Fig. 4B). In addition, complete retention of activity was observed after 24 h at 20 °C. At the temperature above 20 °C its thermostability sharply decreased, varying from 91.0 % to 52.0 % and from 87.0 % to 37.0 % after treatment for 6, 18, and 24 h at 37 °C and 40 °C, respectively. After incubation at 50 °C, the enzyme was almost entirely inactivated.

#### 3.3.2 Determination of the pH dependency of enzyme activity

**Fig. 5:**
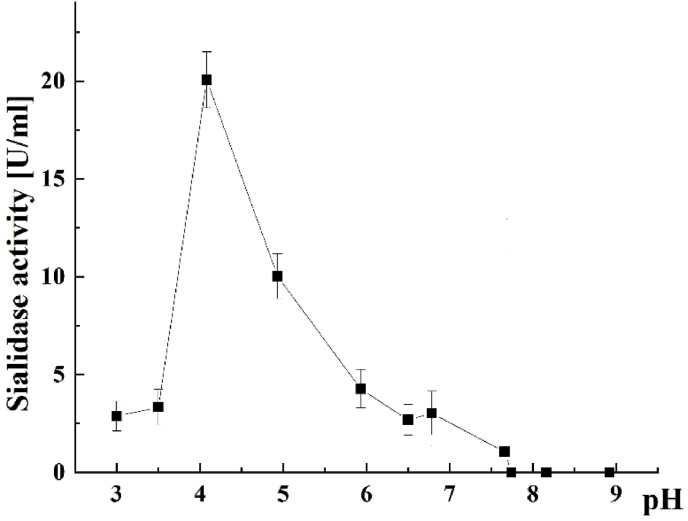
pH-Dependent profile of the *P. griseofulvum* sialidase activity (see M&M). Values are means of three replicates; bars represent the standard deviation. pH turns out statistically significant effect on the enzyme activity (p ≤ 0.05).

Additional information on the activity of purified sialidase from Antarctic *P. griseofulvum* is the determined dependence at different pH values P29 sialidase showed an increase in its activity from pH 3.0, reaching its optimum activity (100 %) at pH 4.0, after which the activity started declining with an increase in pH up to 9.0 (Fig. 5).

Compared to enzyme activity at pH 4.0, it revealed 50.0, 21.20, and 15.25 % activity at pH 5.0, 6.0, and 7.0, respectively. Further, when the pH of the reaction mixture was increased to 7.7, 8.0, and 9.0, the enzyme was completely inhibited. As a result, *P. griseofulvum* enzyme was classified as an acid sialidase.

#### 3.3.3 Substrate specificity

Although the substrate specificity of microbial sialidases has been reported in the literature, little information on substrate specificity is available for fungal enzymes. The studied enzyme was able to hydrolyze glycosylated peptides and sialoglycoproteins as substrates. Table 4 summarizes the activities of sialidase *P. griseofulvum* P29 toward various substrates.

**Table 4:** Substrate specificity of *P. griseofulvum* P29 sialidase.

Substrate	Linkage	Relative enzyme activity [%]
3′-sialyllactose	α(2 → 3)	100
6′-sialyllactose	α(2 → 6)	100
Glycomacropeptide (GMP)	α(2 → 3); α(2 → 6)	60
Horse serum glycoprotein	α(2 → 3); α(2 → 6)	56
Bovine brain gangliosides	α α (2 → 3); α(2 → 8)	36
Fetuin, alpha-2-HS-glycoprotein	α(2 → 3); α(2 → 6)	35
Bovine transferrin glycoprotein	α(2 → 6)	6
Human transferrin glycoprotein	α(2 → 6)	0
Colominic acid (polysialic acid)	α(2 → 8)	0
Monosialoganglioside GM1	α(2 → 3)	0
Monosialoganglioside GM2	α(2 → 3)	0

The relative enzyme activity was determined as the value for sialyllactose was set at 100 %.

The enzyme cleaves sialic acids from the following substrates: 3- and 6-sialyllactose, GMP, horse serum glycoprotein, gangliosides from bovine brain, fetuin and bovine transferrin. It does not release sialic acids from colominic acid, human transferrin, and the gangliosides GM1 and GM2. The addition of Triton X-100 does not affect on the enzyme action towards the ganglioside substrates. With both types of sialyllactose as a substrate, the activity of this enzyme was the highest. This substrate was 1.7-, 1.8-, and 2.8-fold more efficient than GMP, horse serum, bovine transferrin and brain gangliosides, and fetuin, respectively. P29 enzyme was incapable of hydrolyzing colominic acid, human transferrin and the monosialogangliosides GM1 and GM2.

### 3.4 3D model of the sialidase from the fungal strain P. griseofulvum

Comparative analyzes showed 98 % homology between the primary structure of the isolated sialidase from the fungal strain *P. griseofulvum* P29 with the *P. patulum* sialidase. The 3D-model was built based on the 3D structure of *P. patulum* sialidase (KXG51741.1 Neuraminidase) by applying the programs Swiss-Prot, BLAST, RasWin and graphical processing with Swiss-Pdb Viewer.

**Fig. 6:**
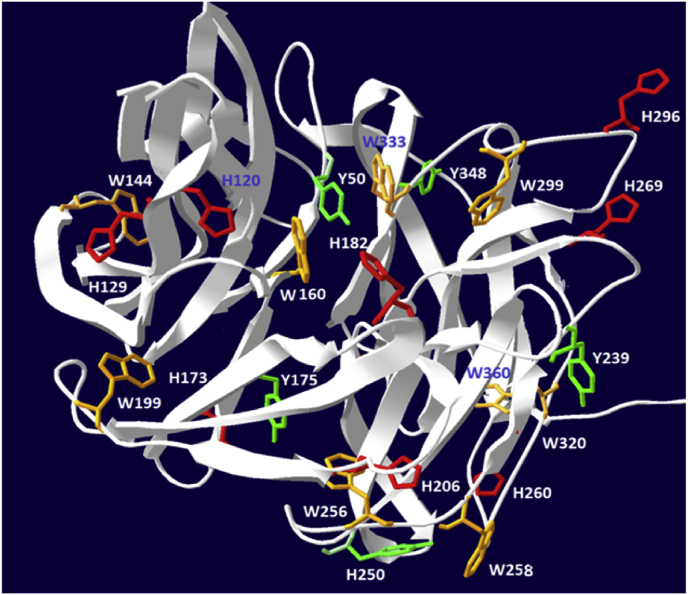
3D-model structure of sialidase from *P. griseofulvum* P29.

The 3D structure of P29 sialidase, presented in Fig. 6, shows the position and different arrangement in the structure of important amino acid residues, such as 5 tyrosines (Y50, Y175, Y239, Y250, Y348), 9 tryptophans (W144, W160, W199, W256, W258, W 299, W320, W333, W360), and 18 phenylalanine.

### 3.5 Fluorescence analysis of P. griseofulvum sialidase

#### 3.5.1 Fluorescence analysis of the structure of sialidase P29

It is known that tryptophan residues (Trps) and tyrosines (Tyrs) are the main AAs, associated with the fluorescence emission of proteins, which makes possible the application of fluorescence spectroscopy, as the most sensitive method to follow a change in the structure of sialidase P29.

**Fig. 7:**
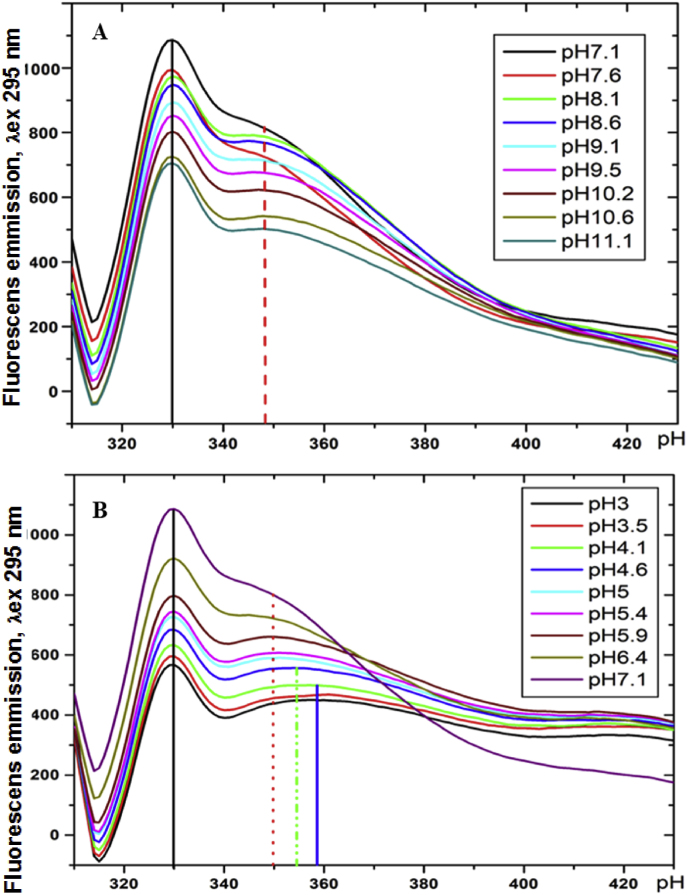
Emission spectra of P29 sialidase at *λ*_ex_ 295 nm: A) in the alkaline region from pH 7.1 to pH 11.1; B) in the acidic region pH 7.1 to pH 3.0.

The presence of 5 Tyrs and 9 Trps allows following the stability of the enzyme at different pH of the solution. The pH dependence of enzyme conformational stability was followed at 20 °C, based on the established high stability of P29 sialidase at low temperatures and full retention of activity after 24 h at 20 °C. The fluorescence properties of the enzyme were analyzed from the emission spectra recorded on a Jasco FP 6600 spectrofluorimeter in the 300 nm–430 nm region after excitation at a wavelength of 295 nm. The absorbance < 0.05 of P29 sialidase was chosen to minimize other effects on the absorbance measured at an excitation wavelength of 295 nm. The emission spectra presented in Fig. 7, dominated primarily by the emission contribution of Trp residues, form two emission maxima (*λ*_max_), at 332 nm and 348 nm.

The presence of these two maxima in the emission spectra for P29 sialidase reflects the different contribution of Trp and Tyr residues, which depends on their location in the enzyme molecule [39,40]. An explanation of the observed maxima is provided by the 3D structural model of P29 sialidase presented in Fig. 6. As the emission maximum (*λ*_max_) at 332 nm in the emission spectra presented in Fig. 7 reflects the emission of “buried” Trp residues in the interior of the molecule. The 3D model of the enzyme shows the indole cores located at the “buried” W144, W256, W320, and W360.

The other emission maximum at 348 nm reflects the emission of the surface-exposed indole rings of W160, W199, W258, W299, and W333, which are mainly responsible for the fluorescence emission. But the location of some of these Trps is near the histidine residues (His) that suppress the emission of the tryptophan ring. The primary structure of the enzyme shows the participation of 14 histidines (H4, H18, H20, H120, H129, H 173, H 182, H 206, H260, H269, H296, H 341, H342, H361), which play an important role in the structure of the enzyme.

**Fig. 8:**
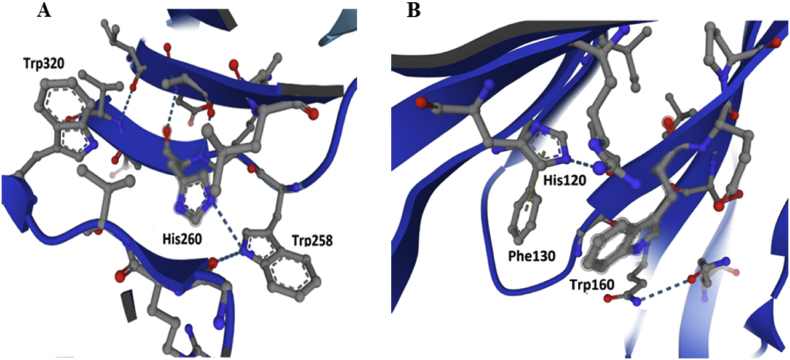
Location of the indole rings of the tryptophan residues relative to the imidazole ring of the side chain of the histidine residues: A) W160 and H182; B) W258 and H260.

Fig. 8 represents the 3D-model of P29 sialidase and the close location of H260 to W258 (Fig. 8A), as well as H182 to W160 (Fig. 8B), suggesting emission “buried” of the indole rings of the Trps by the imidazole rings of the histidines. It can be assumed that the proximity of H206 affects the fluorescence emission of W256, as well as the additional influence on W144, surrounded by two other histidine residues, H129 and H120 (Fig. 6).

#### 3.5.2 Fluorescence analysis of the conformational stability of sialidase P29

**Fig. 9:**
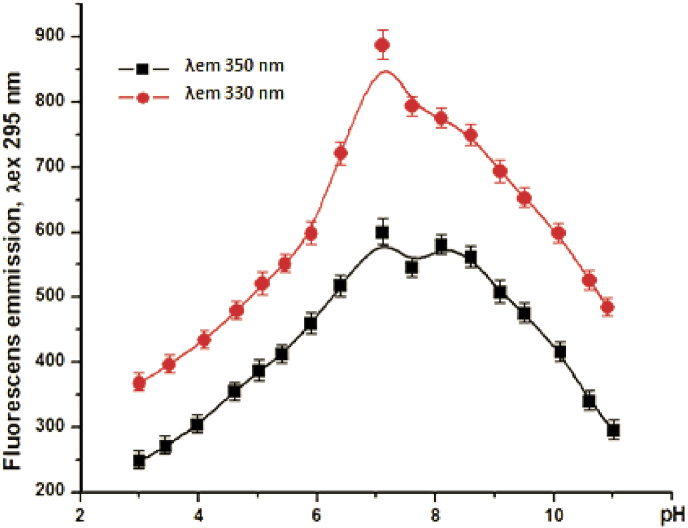
pH-Dependence of the conformational stability of P29 sialidase (from pH 3.0 to pH 11.1), expressed as a change in the fluorescence emission of the enzyme, measured: 1) at *λ*_max_ 330 nm (red) and 2) at *λ*_max_ 348 nm (black). Values are means of three replicates; bars represent the standard deviation. pH turns out statistically significant effect on the conformational stability of P29 sialidase (p ≤ 0.05). (For interpretation of the references to color in this figure legend, the reader is referred to the Web version of this article.)

Additional information on the structure of *P. griseofulvum* sialidase is presented after monitoring its behavior in solution at different pH values of the medium. The conformational change of the P29 sialidase structure is presented after tracking the change in the emission spectra of the enzyme in the alkaline region (pH 7.1 to 11.1) and in the acidic region (pH 7.1 to 3). The results presented in Fig. 9 show different behavior in enzyme titration curves as a function of pH of the solution. Titration curves reflect the ionization of exposed and “buried” Trps. The maximum fluorescence wavelength at *λ*_max_ 330 nm was observed in both alkaline and acidic pH ranges, indicating that the enzyme is pH-stable and does not fully unfold its structure.

In the acidic region, more pronounced changes in the titration curves were observed than in the alkaline region, indicating that the enzyme is much more stable in the alkaline region compared to the acidic region.

Changing the pH in the region from pH 6.5 to pH 8.5 did not change the emission intensity at *λ*_max_ 330 and 348, indicating that no conformational changes occurred in the enzyme structure. Therefore, in this region, the enzyme is pH stable.

## 4 Discussion

The previous results demonstrated that 30 Antarctic fungi possess the ability to produce sialidase [32]. The cold-adapted fungus *P. griseofulvum* P29 has found as a good producer of this enzyme. The novel sialidase would be the only sialidase produced and purified from an Antarctic fungus so far. No reports of sialidases synthesized by non-clinical fungal strains as well as by Antarctic isolates. Cold-adapted fungi have acquired a variety of outstanding biological features arising from harsh environmental conditions including the production of unique enzymes and bioactive molecules with remarkable application potential. Furthermore, enzymes from saprophytic fungi could be a very favorable alternative for scale production and application.

In the present study, an effective laboratory scheme was developed for the purification of P29 sialidase from the fungal strain *P. griseofulvum* on a Q-Sepharose column pre-equilibrated with 0.01–0.2 M potassium phosphate buffer at pH 7.8. The purity of the enzyme was confirmed by 12 % SDS-PAGE and by mass spectrometric analysis, as well as the exact molecular mass of 39924.40 Da, measured by MALDI-Tof/MS analysis. The determined partial AAS by mass spectrometric analyzes confirmed that the isolated enzyme was a sialidase highly homologous in the published amino acid sequence to neuraminidases from *P. griseofulvum*, *P. rubens* Wisconsin 54-1255, and *P. camemberti* (98.3 %, 90.9 % and 91.8 %, resp. respectively). The purified enzyme showed significant activity between 20 and 40 °C with temperature optimum at 37 °C followed by a sharp decrease after 40 °C. As is known, cold-adapted enzymes can effectively catalyze low-temperature reactions and are very temperature-sensitive. Unlike mesophilic and thermophilic enzymes, cold-adapted enzymes show an optimal catalytic temperature generally between 20 °C and 45 °C (Liu et al., 2023). As defined by Margesin and Schinner (1992) enzymes that optimally catalyze at about 30 °C and still have some catalytic efficiency at 0 °C are usually called cold-adapted enzymes. Sialidase P29 should be categorized as a cold-active enzyme.

These data are similar to that reported for the fungal sialidases by *A. fumigatus* [31]. Due to a lack of data on other fungal sialidases, a comparison could be made with bacterial enzymes. A low temperature optimum has been estimated for sialidase by *Listeria monocytogenes* [44], *Pasteurella multocida* B018 [42], and *Oerskovia paurometabola* O129 [43]. At the same time, several bacterial enzymes are more thermostable with a temperature optimum above 50 °C [41,[44], [45], [46]]. Unlike known bacterial enzymes, P29 sialidase demonstrated low thermal stability. The low temperature optimum and reduced enzyme stability confirmed that P29 sialidase is a cold-active enzyme.

The analysis shows that P29 enzyme has a relatively narrower specificity than bacterial enzymes. Bacterial sialidases can catalyze the hydrolysis of terminal sialic acids linked by the α(2 → 3), α(2 → 6), or α(2 → 8) linkages to a diverse range of substrates (de Lederkremer et al., 2022) [47]. Data on the substrate specificity of fungal sialidases are very limited. The enzyme isolated from *A. fumigatus* prefers 3-deoxy-d-glycero-d-galacto-non-2-ulosonates (Kdn glycosides) as substrate compared to *N*-acetylneuraminides (Neu5Ac) [6,48]. P29 sialidase analyzed in this study is most active towards α(2 → 3), and α(2 → 6) bonds. It preferentially cleaved sialic acids from sialosides containing both types of linkages (fetuin, GMP, and horse serum). Kim et al. [49] have reported a similar dependence for *Corynebacterium diphtheriae* sialidase.

The inability of the P29 enzyme to cleave sialic acids from GM1 and GM2 is likely due to steric hindrance [46]. P29 enzyme cannot catalyze the hydrolysis of homopolysaccharides like colominic acid, suggesting little recognition of sialyl α(2 → 8) linkages [50].

The new P29 sialidase is most active in the acidic region, reaching its optimal activity (100 %) at pH 4.0, which does not correspond to the conformational stability of the enzyme. The dependence of the activity of several sialidases on the pH of the medium has been studied very thoroughly, but the conformational stability of the enzyme in alkaline and acidic media has not been studied so far. The presence of Trp and Tyr residues in P29 sialidase allows monitoring of the conformational stability of the enzyme in the pH range from 3 to 11.1 pH by fluorescence spectroscopy.

The conformational changes in the structure of P29 sialidase and the relationship with the activity of the enzyme are reflected by the change in the emission spectra of the enzyme in the alkaline (pH from 7.1 to 11.1) and the acidic (7.1–3) region. The outlined two maxima at 330 nm and 348 nm in the emission spectra for P29 sialidase indicate the different locations of the Trp and Tyr residues in the enzyme molecule. The emission *λ*_max_ of Trp in an aqueous solution is known to be at 350–360 nm, with the shift of the maximum in the emission spectra to shorter wavelengths reflecting tryptophilic side chains in a nonpolar environment or “buried” indole cores of Trp [39,40]. The titration curves reflect the ionization of exposed and “buried” Trps, which is also affected by the quenching of tryptophan emission from nearby histidyl residues. Quenching of tryptophan fluorescence by protonated His residues was first observed by Shinitzky and Goldman [39] and Loewenthal et al. [40].

Information from the 3D location model suggests a “quenching” of the fluorescence emission of the indole rings of W160, W258, and W256 by the proximity of the side chain imidazole rings of H182, H260, and H206, respectively. Also, two other histidine residues, H129 and H120, which surround W144, further influence the emission of its “buried” indole core in the interior of the molecule.

The dominant maximum fluorescence wavelength at 330 nm reflects the emission of “buried” indole cores of W144, W256, W320, and W360, which is hindered by the nonpolar environment in which they reside in the interior of the molecule. This maximum is observed in both alkaline and acidic pH regions, indicating that the enzyme is stable and does not fully unfold its structure at high and low pH values of the medium.

Changes in the second maximum at 348 nm, namely its shift to 350 nm, confirm the conformational changes that occurred in the acidic and alkaline regions, as a result of which the indole cores of the partially “buried” Trps W160, W199, W258, W 299, W333 are fully exposed on the surface of the molecule.

The results of fluorescence studies show that P29 sialidase retains its conformational stability over a wide range of pH 6.5 to 9.0. Important information is the more pronounced changes in the acidic region compared to the alkaline region, indicating that the enzyme is much more stable in the alkaline region than in the acidic region. These results are in good correlation with the pH stability of the two isozymes (NA1 and NA2) produced by the non-pathogenic *Arthrobacter nicotianae* strain. NA1 and NA2 exhibit maximal activity at pH 4 and 5, but both enzymes are conformationally stable in the range of pH 6.5 to pH 9.5 [51].

## 5 Conclusions

Taken together, the results indicate that the fungal strain *P. griseofulvum* P29, isolated from Terra Nova Bay, Antarctica, produces a novel sialidase. The study presents an effective enzyme purification scheme. Based on the low-temperature optimum (37 °C) and low-temperature stability, P29 sialidase can be classified as cold-active. This enzyme is most active toward α(2 → 3) linkages, which have a relatively narrower substrate specificity than bacterial enzymes.

The molecular mass of the enzyme was determined electrophoretically to be about 40 kDa and 39924.40 Da by MALDI-Tof/MS analysis. The partially determined amino acid sequence from MS/MS spectra from mass spectrometric analyzes showed high homology to the published amino acid sequences by genome sequencing of a sialidase enzyme from the fungal strain *P. griseofulvum* P29. The 3D model of the P29 sialidase was built based on its determined primary structure and on the basis of the 3D structure of *P. patulum* sialidase, with which P29 sialidase shows 98 % homology. The purified enzyme retains its conformation when the pH of the solution changes from 6.5 to 8.5 and is much more stable in the alkaline region than in the acidic region.

## CRediT authorship contribution statement

**Aleksandar Dolashki:** Investigation. **Radoslav Abrashev:** Investigation. **Dimitar Kaynarov:** Investigation. **Ekaterina Krumova:** Investigation. **Lyudmila Velkova:** Investigation. **Rumyana Eneva:** Investigation. **Stefan Engibarov:** Investigation. **Yana Gocheva:** Investigation. **Jeny Miteva-Staleva:** Investigation. **Vladislava Dishliyska:** Data curation. **Boryana Spasova:** Data curation. **Maria Angelova:** Methodology. **Pavlina Dolashka:** Conceptualization.

## Declaration of competing interest

The authors declare that they have no known competing financial interests or personal relationships that could have appeared to influence the work reported in this paper.
